# Drought vulnerability assessment and its impact on crop production and livelihood of people: An empirical analysis of Barind Tract

**DOI:** 10.1016/j.heliyon.2024.e39067

**Published:** 2024-10-09

**Authors:** Anika Tahasin, Mafrid Haydar, Md. Sabbir Hossen, Halima Sadia

**Affiliations:** a Department of Urban and Regional Planning, Rajshahi University of Engineering & Technology (RUET), Rajshahi, 6204, Bangladesh; b Department of Urban and Regional Planning, Khulna University of Engineering & Technology (KUET), Khulna, 9203, Bangladesh

**Keywords:** Drought, VCI, TCI, VHI, NDVI, Agricultural production, Livelihoods

## Abstract

North-Western section of Bangladesh is experiencing a protracted decrease in precipitation, irregular rainfall, and the depletion of ground water, which results in water scarcity and extreme dry weather that impedes the production of agricultural commodities and threatens the people's way of life. Analyzing the precipitation deficit and ground water deficit, along with vegetation cover, temperature condition, and the condition of the vegetation is a crucial component of drought vulnerability assessment. Rajshahi Zilla, a region of Bangladesh located in the middle of the Barind tract, is experiencing a severe water shortage. The irregular rainfall, decrease in rainfall, prolonged absence of rainfall and ground water depletion results in drought. This study aims to access the vulnerability of drought by analyzing precipitation rates, ground water depletion levels, temperature condition, vegetation condition and the vegetative droughts to find out the severe condition of droughts and the severe effects of this in the livelihoods of the farmers and their crop production practices. In this case the study focuses on determining NDVI, NDWI, NDMI, VCI, TCI, and VHI. The VHI results show a significant increase in extreme drought conditions from 2013 (4 %) to 2021 (7 %). By conducting a few questionnaire surveys and Focus Group Discussion the present situation of crop production and the livelihoods of people has been analyzed. Almost 18.3 % of farmers have made a permanent move away from agriculture, and 80 % of permanently relocated farmers report an improvement in their quality of life. Nearly 60 % of farmers believe that the construction of deep ditches has enhanced their crop yield. Once again, more than 20 % are in the same predicament as previously, with over 19 % reporting that deep irrigation has lowered agricultural yield. Comprehending the potential consequences of drought will enable planners and decision-makers to implement mitigation measures aimed at improving the communities' ability to manage drought risk. After analyzing data, it has been found that Rajshahi is facing a critical drought problem, which has led to water scarcity, severely affecting agricultural production and livelihoods.

## 1 Introduction

Bangladesh is one of the most susceptible to natural calamities among all developing countries globally. This is because it has an unpredictable climate, a low, flat topography, a unique hydrogeological setting, and a varied, complex geomorphology. Almost every year, Bangladesh has been affected by severe extreme weather events like droughts, floods, tropical cyclones, and storm surges, which have caused a lot of damage and loss of life [1]. Bangladesh is one of the countries with the largest number of people living in cities. It has about 940 people per square kilometer, but each person earns about US$235 a year. More than 40 % of the people in the country are living in poverty. Bangladesh is especially prone to disasters because it has a high population density, widespread poverty, and significant social injustice. It also has a climate that changes a lot in space and time, terrible weather, few financial resources, and weak infrastructure [2].

Drought is a common occurrence in many parts of Bangladesh, particularly in the northwestern region. Drought is one of the biggest problems in farming because it hurts crop growth. Bangladesh is an agricultural country struggling to adapt to climate change, a growing population, and the need for food security. Climate change predictions say that drought will be a big problem in the western part of Bangladesh. In his study [3], said that Bangladesh would have more frequent and severe droughts in the near future. Bangladesh has had more and worse droughts in recent years because of changes in how the land is used.

Drought is the most destructive climate hazard, affecting hydrology, meteorology, ecology, and society. Drought can cause significant economic losses as well as a variety of negative effects on human life [4]. Drought is a stochastic, multidimensional climatic hazard that is undeniably one of nature's most complicated phenomena. It is an inevitable occurrence, an inevitable component of the climate. It usually spreads over wide areas at a gradual pace. Natural mechanisms that are difficult to perceive and influence govern its occurrence, intensity, gravity, duration, and spatial expanse. Drought has disastrous and sometimes irreparable effects on local communities and agro-ecosystems all over the world [5]. Drought is a critical global issue impacting economies, societies, and ecosystems across the world. Natural climate variability plays a role in drought events, but human activities such as deforestation, overuse of water resources, and climate change amplify their frequency and severity [6]. Climate change, in particular, disrupts precipitation patterns and increases evaporation rates, worsening drought conditions. The consequences are severe, leading to substantial agricultural losses, water shortages, and forced migration, which in turn exacerbate food insecurity and economic instability. Regions like Sub-Saharan Africa, Australia, and parts of the Americas frequently suffer from devastating droughts, underscoring the urgent need for sustainable water management and climate adaptation strategies [7]. Tackling the root causes and mitigating drought impacts is essential for maintaining global environmental and socioeconomic stability [8]. Drought risk is on the rise globally. Drought is generally on the rise worldwide between the 20th century and both of the coming centuries [9]. The fact that the geographic patterns of growing and decreasing dryness during the middle and late twenty-first centuries appear to be relatively comparable is more intriguing, though. Accordingly, by the end of the century, there may be a greater frequency of sporadic and erratic severe drought episodes, which could affect human activity, dry up the rainforest ecosystem, and alter the entire global carbon cycle [[10], [11], [12]].

The severity of a drought varies on several factors. Droughts are caused when there is an insufficient amount of precipitation [13] says that droughts happen when the land can't hold enough water or rain. The main cause of droughts in plants is a lack of water in the soil during the growing season [4]. High temperatures and a lot of evaporation and condensation cause drought in plants. The most common natural disaster is drought, which hurts crops and people's way of life. Drought hurts the health and output of plants. Low rains, high temperatures, and evaporation of sweat can all cause it.

The Barind region is one of the area's most severely affected by the drought. Because of how it affects GDP and food security, drought severity is becoming more of a threat to farming output in the Barind tract [14]. High temperatures, minimal rain, and little soil moisture storage are all characteristics of this area. Climate change is especially dangerous for the crops grown in the Barind area. One of the main reasons for this changing climate is that many weather events are happening, like flash storms, droughts, temperature rises, and not enough rain (Rashed et al., 2019). Recently, drought has become one of the Barind areas' most important weather problems. The main causes of the drought in the northwestern part of the country are the loss of ground water, changes in how it rains, and rising temperatures in the Barind plains. The environment, agricultural practices, and socio-economic issues are all negatively impacted by drought. According to the researcher, the severity of the drought leads to a drop in agricultural output, poor environmental management, less groundwater recharge, and other problems [15,16]. Consequently, one of the most important tasks at hand right now is to look for possible solutions that will lessen the impact of the drought.

Due to rising temperatures, the area is getting less rain over time. The normal annual rainfall in Rajshahi is only 1625 mm, which is a lot less than the average of 2550 mm for the whole country [17]. Rainfall is very important for agriculture, which is the main economic driver in Rajshahi Division. Because of this, changes in the way it rains could have a big effect on the agricultural business in the area. Crop output is affected directly or indirectly by things like high temperatures, drought, strange rain patterns, and global warming. These climate changes have severely affected crop yields [18]. Because of this, farmers are not urged to do things related to farming. Most of the people leave their jobs because of a drop in production. The switch from making rice to making other crops. As a consequence of this, they are unable to make sufficient earnings. In general, they focus on foods that need less water. That's how they change and move to a new way of life.

Bangladesh, being susceptible to several natural hazards, finds GIS and RS approaches highly beneficial for quantifying the magnitude of these hazards [19,20]. Satellite images have been used to find signs of drought. Because satellite images have high-resolution data, they have been used to get a better idea of how the drought is affecting the area under study [21]. Drought indices are one of the best ways to use satellite data to find out where droughts are likely to happen and how bad they are in different places [22]. The Vegetation Condition Index (VCI), the Thermal Condition Index (TCI), and the Vegetation Health Index (VHI) are three ways to tell if there is a drought [23]. The Vegetation Health Index (VHI) is a hybrid index that combines two different measurements into a single value: the Vegetation Condition Index (VCI) and the Thermal Condition Index (TCI) [16].

From 1984 to 2003, researcher showed water and climate statistics for the Aravalli region. The NDVI, NDWI, NDMI, VCI, TCI, and VHI are all used to measure how dry plants are. VHI is a better way than VCI and TCI to track drought in plants. The effects of drought on climate, water, and plants in the Aravalli region were studied [24].

The researcher said that the Standardized Precipitation Index (SPI) method has been used to measure weather drought [25]. The SPI is used to see if there is a link between droughts and floods caused by global warming and the amount of global warming. In the same way, temperature records are used to look for possible connections between global warming and the climate of the research site. Spatial maps of Ground Water drought for different threshold values show that most of the study area is regularly affected by drought, but the northern half is not. The main cause of Ground Water droughts is the loss of Ground Water from the subsurface groundwater, which can be seen in the Ground Water hydrographs for most of the observation wells during the study.

Kafy et al., 2023 use Landsat satellite images from 1996 to 2031 to look at how vulnerable Barind Tract is to drought and make a prediction. The plant Health Index (VHI) takes into account the amount of water in the soil, the temperature condition index, the plant condition index, the vegetation health index, and the normalized difference vegetation index (NDVI). Based on the features of VCI and TCI, Cellular Automata (CA) and Artificial Neural Networks (ANN) were used to find DS, and predictions were made for 2026 and 2031.In their study, Habiba and her colleagues also pointed out that dryness is a serious problem. Farmers in Northwestern Bangladesh, which is the most drought-affected part of the country, are using a variety of methods to deal with the effects of drought. Adaptation options help farmers meet their goals of food, income, and livelihood security even though the weather is changing.

The objective of this study is to assess the susceptibility of individuals to drought by analyzing various indicators, including Vegetation Condition Index (VCI), Temperature Condition Index (TCI), and Vegetation Health Index (VHI), in addition to examining livelihood patterns and shifts in agricultural practices. The study commences with a comprehensive elucidation of the drought and its profound ramifications, along with the factors that contribute to the severity of the drought. Following the introduction, there is a section titled "Methodology" that provides a concise overview of the study and the techniques employed to assess indicators related to drought. The results and discussion section provides a comprehensive explanation of the findings, relevant factors, various agricultural crops, and the livelihood strategies employed by farmers. Ultimately, the current study concludes by providing a synopsis, as well as recommendations for future investigations.

The main objective of our study was to assess the vulnerability of the northwestern region of Bangladesh, particularly Rajshahi zilla, to drought. We assessed the quantity of vegetation and its corresponding temperature. The primary objective of this study is to ascertain the livelihoods of those residing in the Barind tract and determine the associated costs. This analysis will be conducted by examining the fluctuations of several drought indicators between consecutive years. This study distinguishes itself from previous ones by delving more extensively into the underlying reasons of prolonged droughts and erratic rainfall patterns in the region. Further research is required to determine the impacts of additional potential factors contributing to drought, such as alterations in land utilization, meteorological patterns, and water management practices. There is a lack of extensive research on the specific factors that contribute to the fluctuations in aquifer stress, leading to water shortages in certain areas of the region. Furthermore, it lacks a comprehensive understanding of the strategies employed by farmers and other individuals in the region to mitigate the effects of drought. For instance, further research may investigate the efficacy of existing adaption measures and identify potential barriers to their implementation. This would facilitate the development of targeted strategies to assist individuals in coping with drought conditions.

## 2 Materials and Methodology

### 2.1 Description of the study area

**Fig. 1:**
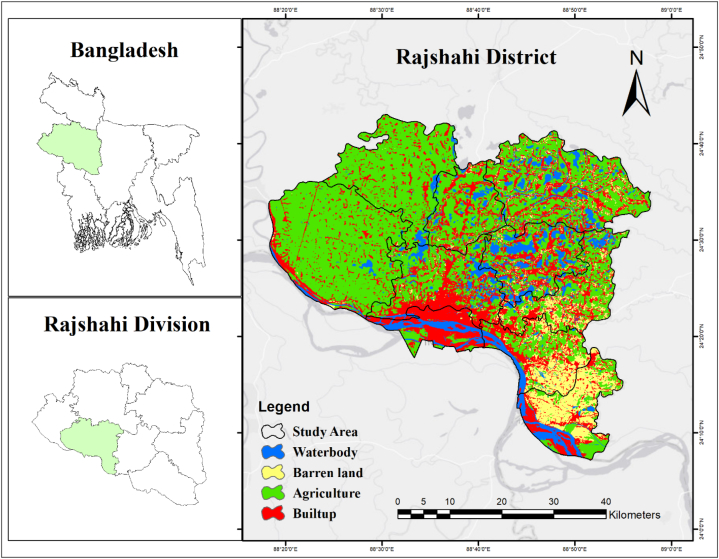
Study area map (Rajshahi district).

The geographical location of the study area is 24.3733° N, 88.6049° E. The study was mostly done in the Barind part of the Rajshahi district. Drought and a lack of water are significant problems in the north-west of the country. There are seven upazilas in the Rajshahi district: Godagari, Paba, Rajshahi Metro, Charghat, Puthia, Durgapur, Mohanpur, Baghmara, and Tanore. The LULC of the study area depicts, there are 7.30 %, 21.43 %, 41.88 %, 29.39 % of Waterbody, Baren Land, Agricultural Land and Builtup areas respectively (Fig. 1). This area has a typical dry climate with high temperatures. The place chosen is known for its agricultural areas. Due to the arid conditions, it's hard to grow crops in this area during the summer.

### 2.2 Dataset descriptions

For the year 2013, 2017, 2021 (Landsat 8 Imageries; cloud cover <10 %) were downloaded from USGS Earth Explorer (Table 1). After collecting the images, preprocessing was carried out. After collecting images, preprocessing was carried out. After receiving the satellite images, data processing was carried out to arrange and prepare the data for future study, which included selecting the bands and data, among other things. Finally, the data gathered from satellite images was processed by digitizing it. Agricultural drought was determined by analyzing NDVI, NDWI, NDMI, TCI, VCI, and VHI values over a variety of Landsat OLI 8 bands.

**Table 1:** Dats Sources.

Data acquired	Scene ID	Sensor	Cloud coverage
01.01.2013-01.12.2013	LC08_L2SP_138043_20131129_20210912_02_T1_SR_B3	Landsat 8 OLI/TIRS C1 Level-2	<10 %
01.01.2013-01.10.2013	LC08_L2SP_138043_20131129_20210912_02_T1_SR_B4		
01.01.2013-01.10.2023	LC08_L2SP_138043_20131129_20210912_02_T1_SR_B5		
01.01.2013-01.10.2013	LC08_L2SP_138043_20131129_20210912_02_T1_SR_B6		
01.01.2013-01.12.2013	LC08_L2SP_138043_20131129_20210912_02_T1_ST_B10		
01.01.2017-01.12.2017	LC08_L2SP_138043_20171121_20210905_02_T1_SR_B3	Landsat 8 OLI/TIRS C1 Level-2	<10 %
01.01.2017-01.12.2017	LC08_L2SP_138043_20171121_20210905_02_T1_SR_B4		
01.01.2017-01.12.2017	LC08_L2SP_138043_20171121_20210905_02_T1_SR_B5		
01.01.2017-01.12.2017	LC08_L2SP_138043_20171121_20210905_02_T1_SR_B6		
01.01.2017-01.12.2017	LC08_L2SP_138043_20171121_20210905_02_T1_ST_B10		
01.01.2021-01.12.2021	LC08_L2SP_138043_20211116_20210315_02_T1_SR_B3	Landsat 8 OLI/TIRS C1 Level-2	<10 %
01.01.2021-01.10.2021	LC08_L2SP_138043_20211116_20210315_02_T1_SR_B4		
01.01.2021-01.10.2021	LC08_L2SP_138043_20211116_20210315_02_T1_SR_B5		
01.01.2021-01.10.2021	LC08_L2SP_138043_20211116_20210315_02_T1_SR_B6		
01.01.2021-01.12.2021	LC08_L2SP_138043_20211116_20210315_02_T1_ST_B10		

From January to March 2023, field surveys and Participatory Rural Appraisal were used in Rajshahi to get first-hand information. The main information was gathered using a structured questionnaire that had already been tried. Primary and secondary data for this study are gathered by questionnaire surveys, in-person interviews, and physical observations. Once more, the major data was gathered using a few PRA tools, including the Focus Group Discussion, Seasonal Calendar, and Resource Map. There almost 50 farmers were surveyed to conduct the study. The original data were gathered using the snowball sampling approach from this demographic. After that, the farmers were questioned, and a variety of data was gathered. Finally, SPSS and Microsoft Excel were used to handle and analyze the data. Several journal papers, articles, newspaper stories, and so on were used to get secondary data. Major questions are- (i) How is the change in the crop production status? (ii) crop switching status (iii) Seasonal migration time period (iv) How long you have changed your job? (if permanent) (v) How is the change of living standard?

### 2.3 The estimation of NDVI

In the study conducted by Ref. [5], it was shown that the existence of a resilient and varied vegetation cover is of utmost importance in preserving the thermal balance of terrestrial surfaces and alleviating the potential for exacerbating drought conditions within a specific area. The Normalized Difference Vegetation Index (NDVI) is a metric that quantifies the presence of vegetation in different atmospheric areas, encompassing the gaps between clouds and the Earth's surface. Its purpose is to provide an estimation of the degree of vegetation coverage within a specific geographical area.

The Normalized Difference Vegetation Index (NDVI) measures vegetation health and production and is used to assess drought. Satellite plant NIR and red reflectance data are used to calculate NDVI [[26], [27], [28]].

$$NDVI=\frac{Rnir-Rred}{Rnir+Rred}$$

The normalized difference vegetation index (NDVI) is a metric used to evaluate drought by gauging the state of plant life and productivity. The red and NIR reflectance readings from satellites are used to figure out the NDVI. The thermal radiation efficiency of the land area is measured by its emissivity. With the Normalized Difference Vegetation Index (NDVI), it can measure the state of health and growth of plants. The NDVI is based on the chlorophyll in plants, which shows how healthy they are. With more-green, the NDVI will increase. Thus, the NDVI can track how plants grow and how much food they produce. This method is often used to check on the health of crops, forests, fields, and other types of plant life [29].: The NDVI is usually calculated with the help of sensors on satellites or aerial platforms that measure the reflectance of the earth's surface in the visible and near-infrared bands. There were three groups of NDVI values: sparse vegetation, middling vegetation, and dense vegetation. Drought can reduce the amount of cover and the productivity of plants, which makes NDVI readings go down. We can figure out how drought affects the health and productivity of plants by keeping track of NDVI readings over time.

**Table:** Classification of the NDVI based on the NDVI range.

NDVI range	NDVI class
0.03-0.30	Sparse
0.30-0.50	Moderate
≥ 0.50	Dense

### 2.4 The estimation of NDWI

The NDVI, NDWI, and NDMI can be used to locate and monitor a region experiencing drought. Drought intensity is measured by a number of indicators, the most prominent of which are plant stress, water availability at the surface, and insufficient soil moisture saturation [30]. Researchers found that drought monitoring and assessment could benefit from these plant and water characteristics [31,32].

NDWI looks at how much water is in plants to determine how stressed agriculture is [33]. uses the following equation to calculate it. To compute the NDWI, take the difference between the green and near-infrared bands and divide it by the total of the two bands.

$$\text{NDWI}=\frac{RGreen-Rnir}{RGreen+Rnir}$$

where GREEN is the green reflectance of the multispectral image and NIR is the near infrared reflectance of the multispectral image.

Also, NDWI values can be anywhere from −1 to +1. Positive numbers mean that there are water features, which is desirable. On the other hand, dirt and plants usually have zero or negative values, so they are pushed down. In agricultural stress studies, low NDWI readings could suggest that the crops are under water stress, which could affect their growth and yield. Monitoring NDWI over time can show where crops are getting too little water and provide insights about irrigation and water use in those areas [34].

### 2.5 The estimation of NDMI

NDMI is a trustworthy measure for gauging the interplay between precipitation, soil moisture, and plant canopy water content. the year [35]. NDMI is an essential tool for drought monitoring because of its higher spatial resolution, which is required to track plant water content through the measurement of reflectance.

[36] state that NDMI helps detect the liquid content from plants that indicates the state and level of disturbance in the forest. The NDMI is a remote sensing index that measures how much water is in plants. The estimate is based on the reflectance values of NIR and midinfrared (MIR) wavelengths, which are sensitive to water in plant tissues. The NDMI is found with the help of the following equation.

$$\text{NDMI}=\frac{Rnir-RSWIR}{\;Rnir-RSWIR}$$

The range of the NDMI is from 1 to 1, with negative values meaning dry conditions and positive values meaning wet conditions. When the NDMI number is close to zero, it indicates that there is a moderate amount of water. NDMI is often used to figure out how productive and healthy a plant is in general and to keep an eye on crop wetness stress. It can be used to identify dry areas and track how the amount of water in plants changes over time. NDMI is often combined with other remote sensing measures, like the NDVI, to get a more accurate picture of how crops are doing [37].

### 2.6 The estimation of LST

The BT region's surface temperature distribution was determined using Landsat thermal bands and Plank's law [38].

Lλ = MLWQcal + AL

Where L represents the radiation from the spectrum, ML represents the multiplicative rescaling factor that is specific to the band based on the metadata, and AL represents the specific z rescaling factor that is also specific to the band based on the metadata. "Quantized and calibrated" refers to as "qcal," which is an abbreviation for "quantized and calibrated," and it refers to the standard product pixel values. The following equation can be used for the purpose of translating spectral radiation to temperatures that are stated in kelvin:

$$T=\frac{K2}{\ln\;\left(\frac{K1}{L\lambda }+1\right)}$$

Where K1 and K2 are band-specific thermal conversion constants derived from the metadata, T is the satellite's brightness temperature (K), and L is the TOA's spectral radiance (W/m2 ster m), and Temperature is derived in 'Kelvin (A),' which was converted into 'degree Celsius (B)' making use of the following equation:

B = A−273.15

Therefore, the final LST equation is as follows-

$$\text{LST}=\frac{\text{Ti}}{1+\left(\;\lambda *Ti/\left(\;\rho \right)\right)*\ln\;\varepsilon }$$

The LST was used to calculate TCI values where the TCI equation is

$$\text{TCI}=\frac{LST-LSTmin}{LSTmax-LSTmin\;}\times 100$$

### 2.7 The estimation of vegetative drought indices VCI, TCI, VHI

Kogan (1990) claims that VCI enables describing vegetation, assessing geographic and temporal changes in vegetation, and determining the influence of weather on vegetation. According to Kogan (1995), VCI is a reliable tool for detecting drought and gauging its severity, duration, and negative effects on vegetation.

In addition to mentioning VCI, TCI, and VHI as important drought markers, by controlling transpiration and evapotranspiration, VCI affects soil moisture and plant water status. Vegetation cover changes that minimize evaporative cooling can enhance TCI during dry seasons, especially when the steam potential is low. The susceptibility to drought is exacerbated by VHI, in particular, due to the increased frequency of severely hot weather occurrences.

A rise in the number of places reporting severe, extreme, or moderate drought was observed across all indices, whereas the number of places reporting mild or no drought fell considerably. The percentage of VCI space experiencing severe or extreme drought increased between 2013 and 2021, whereas the percentage experiencing mild or no drought decreased. However, severe and extreme drought areas increased between 2013 and 2022 in TCI, while mild and no drought areas dropped dramatically. Similar to VHI, regions experiencing severe and acute drought have expanded while areas experiencing mild or no drought have shrunk.

Drought could be indicated by low VCI readings, which could suggest stressed or dried-out plant life. When the TCI is high, it may suggest that there is a risk of drought due to increased evaporation and moisture stress. Since the VHI takes into account the effects of heat stress, soil moisture related to vegetation, and plant desiccation, it provides a more accurate estimate of drought severity. As low VHI values are indicative of dry or stressed vegetation, a rise in the severity of the drought may be indicated by this trend. Drought conditions can become even more severe if precipitation is scarce, evaporation rates are high, and temperatures are high [39].

The vegetative health indicator (VHI) is used to track and predict how agricultural DS will affect plant life. Drought-related issues can be identified with the help of the VHI, which can then be used to develop adaptive and mitigating measures. The VHI also shows how well plants are able to adjust to adverse conditions. The VHI is heavily influenced by both the temperature (TCI) and vegetation (VCI) conditions [24]. The VCI, is a method frequently used in agricultural contexts to assess plant health [40].

There is a close relationship between the weather and plant drought. In NDVI, the weather component is dwarfed by the more prominent biological one. However, VCI can differentiate between weather-related NDVI fluctuations that last only a few days and those that last for years [27,28]. VCI, on the other hand, varies and reflects relative changes in vegetation status from extremely poor to good [[26], [27], [28]], while NDVI indicates seasonal vegetation dynamics. On the other hand, the Temperature Condition Index shows how the temperature has changed in relation to the ambient light.

$$\text{VCI}=\frac{NDVI-NDVImin}{NDVImax-NDVImin\;}\times 100$$

Plant stress from heat and dryness is brought on by low TCI. When temperatures are high and rainfall is light, minimum TCI values occur [41]. Since TCI distinguishes between drought and non-drought conditions, VCI is unable to forecast drought. According to Ref. [42], the TCI determines whether excessive moisture or dryness results in vegetative stress. TCI forecasts the onset, duration, air temperatures at the surface, and rooting depth of droughts [43].

$$\text{TCI}=\frac{LST-LSTmin}{LSTmax-LSTmin\;}\times 100$$

Higher VCI values are associated with healthy moisture conditions and unstressed vegetation. The utilization of TCI data analysis has the potential to effectively monitor subtle alterations in the well-being of vegetation resulting from thermal stress, as demonstrated by Kogan's studies conducted in 1995, 2001, and 2002. The Vegetation Condition Index (VCI) and Temperature Condition Index (TCI) are utilized to differentiate vegetation based on different levels of moisture and temperature. On the other hand, the Vegetation Health Index (VHI) provides a comprehensive assessment of overall vegetative health [[26], [27], [28]].

VHI = 0.5∗(VCI) + 0.5∗(TCI)

## 3 Results & discussions

### 3.1 Temporal analysis of different drought parameters

**Fig. 2:**
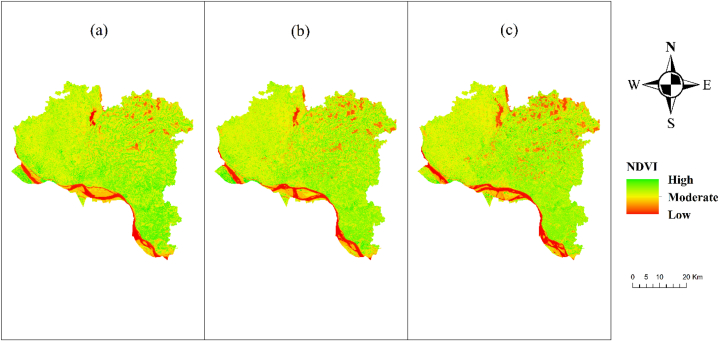
NDVI map of Rajshahi District (a) 2013, (b) 2017, (c) 2021.

In Fig. 2, the results illustrate that in 2013 there was dense vegetations, and in 2017 the amount of dense vegetation had decreased, while the amount of sparse and moderate vegetation had increased. In 2021 the amount of dense vegetation decreased and the number of moderate and sparce vegetation increased. The severity of drought in the Barind region was assessed by employing NDVI time series analysis, which served as a crucial indicator. The findings of the study indicate that there was a higher concentration of dense vegetation in the year 2013. However, in the year 2017, there was a decrease in the amount of thick vegetation, accompanied by an increase in the presence of sparse and moderate vegetation. In the year 2021, there was a reduction in the extent of dense vegetation, accompanied by an increase in the prevalence of sparse and moderate vegetation. The ongoing decline of vegetative areas can be attributed to rising temperatures and reduced precipitation. From the field survey, it was found that farmers are shifting to crops requiring less water, such as vegetables. As rice, wheat or other main crops require more water, they are now shifting their focus. On the other hand, the ground water level in going down, the soil moisture is also decreasing, and in the summer season, due to extreme heat, surface water is also not available. The Normalized Difference Vegetation Index (NDVI) exhibited its highest value in the year 1990, followed by a decline leading to its lowest value in the year 2020. The research findings indicate that the year 2020 saw the most arid conditions, while the year 1990 experienced the highest levels of precipitation [6]. The observed decrease in vegetation NDVI value can potentially be attributed to the escalating intensity and frequency of drought in the research area. This finding aligns with the notion that drought-induced stress hampers photosynthetic activity [18], leading to increased mortality rates and reduced plant recruitment and seedling establishment. According to a study conducted in the north-western region of Bangladesh, it was observed that the prevailing water channels, including rivers and canals, were more impacted by drought conditions. This phenomenon has resulted in a significant decline in the overall functioning of the ecosystem [2].

The research area is particularly prone to drought severity due to the hot climate brought on by irregular rainfall patterns, temperature swings during the day and night, and a decline in surface water bodies. Temperatures rise as the number of erected structures increases. Drought is exacerbated by rising temperatures. On the other side, urban growth has resulted in an increase in construction, which tends to fill up water bodies, potentially causing drought conditions in the future. According to Rahaman et al., the annual average precipitation in Bangladesh's northwestern region declined from 151.50 mm in 1994–2003 to 138.09 mm in 2004–2013. Drought conditions are driven by decreasing rainfall and rising temperatures.

**Fig. 3:**
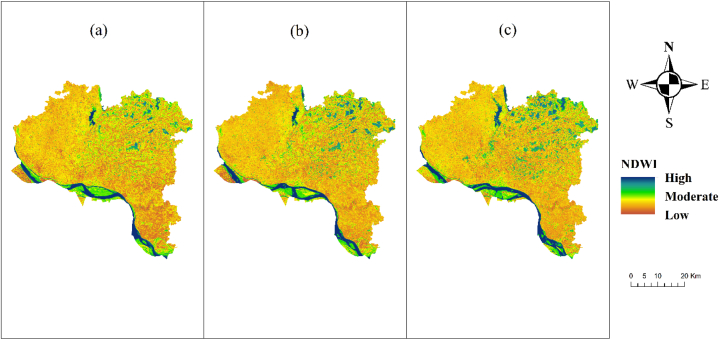
NDWI map of Rajshahi District (a) 2013, (b) 2017, (c) 2021.

One of the most commonly mentioned causes of droughts is the depletion of surface water supplies. The higher index values for the study area have decreased between 1996 and 2021, as shown by the NDWI evaluation. According to the NDWI, most bodies of water have shrunk in size in various locations (Fig. 3). Since regions with low NDWI values are more prone to severe drought, and regions with high NDWI values indicate low or no drought. Lower precipitation and higher average yearly temperatures may explain the decline observed in NDWI values over the past decade. Our findings suggest that declining precipitation and increasing annual temperature may have a negative effect on NDWI values in the regions we analyzed.

**Fig. 4:**
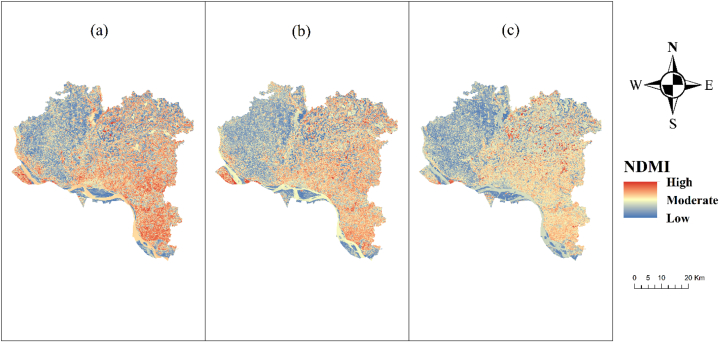
NDMI map of Rajshahi District (a) 2013, (b) 2017, (c) 2021.

Drought can be identified by high rates of evaporation, shifts in the moisture balance, and an escalation in rainfall variability. Changes in land usage and the expansion of built-up areas can both impact the level of soil moisture. As the vegetation coverage decreases, the plant population likewise declines, resulting in a fall in soil moisture levels (Fig. 4). Both transpiration and evapotranspiration impact the soil's moisture content and the water status of plants. An increase in temperature also contributes to the deterioration of the water quality in the soil. The data demonstrate a significant decrease in soil moisture in the Rajshahi district.

**Fig. 5:**
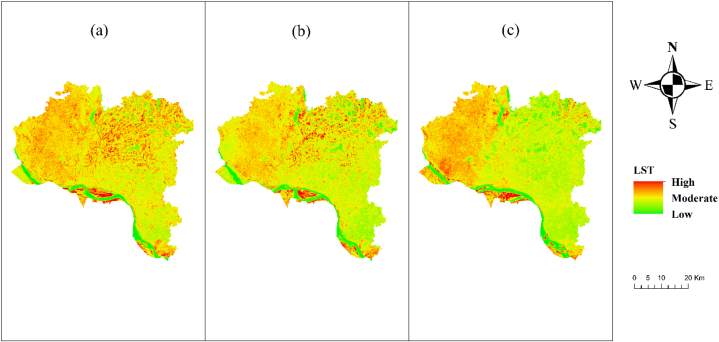
LST map of Rajshahi District (a) 2013, (b) 2017, (c) 2021.

Fig. 5 depicts the temporal variations in Land Surface Temperature (LST) throughout the Rajshahi district over the years 2013, 2017, and 2021. The analysis reveals that the land surface temperature (LST) in the northwestern region of the research area has significantly increased in 2021 compared to 2013. Ultimately, these factors exert a substantial influence on soil moisture levels. In the southeastern area, the Land Surface Temperature (LST) is very consistent in all years.

### 3.2 Variation in VCI, TCI and VHI

**Fig. 6:**
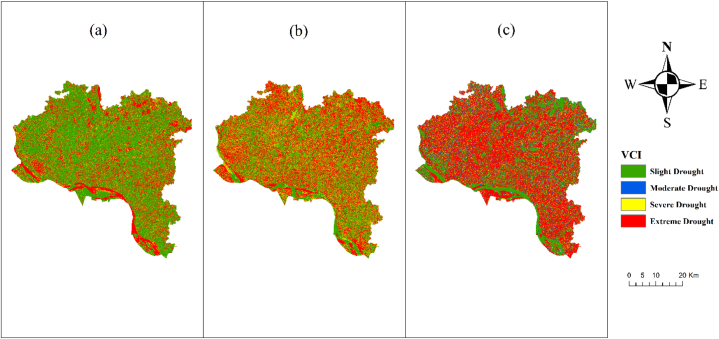
VCI map of Rajshahi District (a) 2013, (b) 2017, (c) 2021.

The severity of the drought persists for the entirety of the year. From 2013 to 2021 (Fig. 6), there was an observed increase in the occurrence of severe and intense drought events as measured by the Vegetation Condition Index (VCI), whereas the occurrence of moderate and no drought events exhibited a reduction. The occurrence of severe drought is attributed to the combination of rising temperatures and declining vegetation. The quantity of structures is also increasing. Certain infrastructure developments, such as the construction of new motorways and other construction projects, have adverse effects on the surrounding vegetation, leading to its demise. The increase in VCI can be attributed to this factor. According a study undertaken by Ref. [40], the VCI is a metric that is frequently utilized for the purpose of describing the vegetation status in agricultural fields. According to Ref. [44] research from 2022, low levels of TCI create plant stress, while high values of VCI contribute to stable, unstressed plant growth (high temperatures and drought). The NDVI values that were calculated to be the highest and lowest for the period of 2013–2021 were used in the process of determining the features of the VCI. A low VCI indicates that the vegetation is stressed or dry, which may be an indicator that drought conditions are present.

**Fig. 7:**
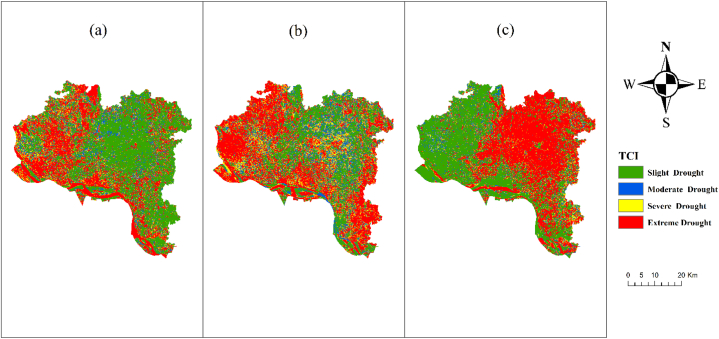
TCI map of Rajshahi District (a) 2013, (b) 2017, (c) 2021.

Between 2013 and 2021, the data consistently indicated a significant deterioration in regions experiencing severe, extreme, and moderate drought, whereas regions with mild or no drought experienced substantial improvement (Fig. 7). From 2013 to 2021, the frequency of severe and extreme droughts in TCI increased twofold, whereas the occurrence of moderate and no droughts decreased significantly. Due to a huge rise in temperature, the drought in the area is getting worse. In the middle of summer, the temperature stays the same. Extreme heat and the loss of production and crops are both caused by the rise in temperature. On the other hand, when it rains less and there are fewer plants and new buildings, the temperature goes up, which tends to make the TCI number go up. Temperatures may rise because of factors like climate change, global warming, haphazard development, and the loss of plants and aquatic life in the study area [45]. When temperatures rise, precipitation, evaporation, and transpiration all go down. This causes hydrological and farming dryness, which can lead to drought in TCI. On the other hand, healthy vegetation changes the temperature balance of land surfaces and makes it less likely that a drought will cause a region to become highly dry [5].

**Fig. 8:**
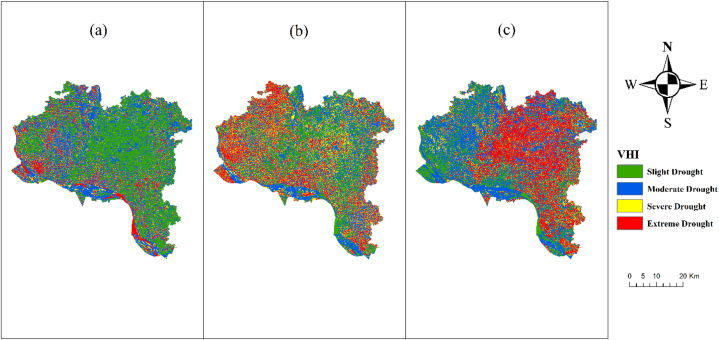
VHI map of Rajshahi District (a) 2013, (b) 2017, (c) 2021.

From 2013 to 2021 (Fig. 8), empirical data continuously showed a considerable worsening of drought conditions in regions experiencing severe, intense, and moderate drought, whereas places with mild or no drought showed significant improvement. Between 2013 and 2021, there was a significant rise in the prevalence of severe and intense droughts, as evidenced by a twofold increase in their frequency within the Vegetation Health Index (VHI). In contrast, there was a significant decline in the frequency of moderate and no droughts throughout the same period. The worsening of the drought in the region can be attributed to a substantial rise in temperature. Throughout the summer season, the temperature remains consistent. Rising temperatures are the cause of both the occurrence of extreme heat events and the subsequent adverse effects on productivity and agricultural produce. On the other hand, during times of decreased rainfall and less plant growth caused by urbanization, the temperature rises, resulting in an increase in the VHI value. The Vegetation Condition Index (VCI) regulates the processes of transpiration and evapotranspiration, which in turn have an impact on the levels of soil moisture and the water status of plants. During periods of reduced vegetation cover that lead to a decrease in evaporative cooling, the Temperature Condition Index (TCI) may increase, especially when the moisture content in the atmosphere is low. Vulnerability Health Index (VHI) has a significant role in the occurrence of extreme heat events and their impacts on human health and well-being, thereby heightening its vulnerability to drought conditions. Droughts resulting from fluctuations in Vegetation Health Index (VHI) lead to agricultural devastation and increased food costs for both farmers and individuals with limited financial resources.

### 3.3 Analysis on drought effects on crop production

**Fig. 9:**
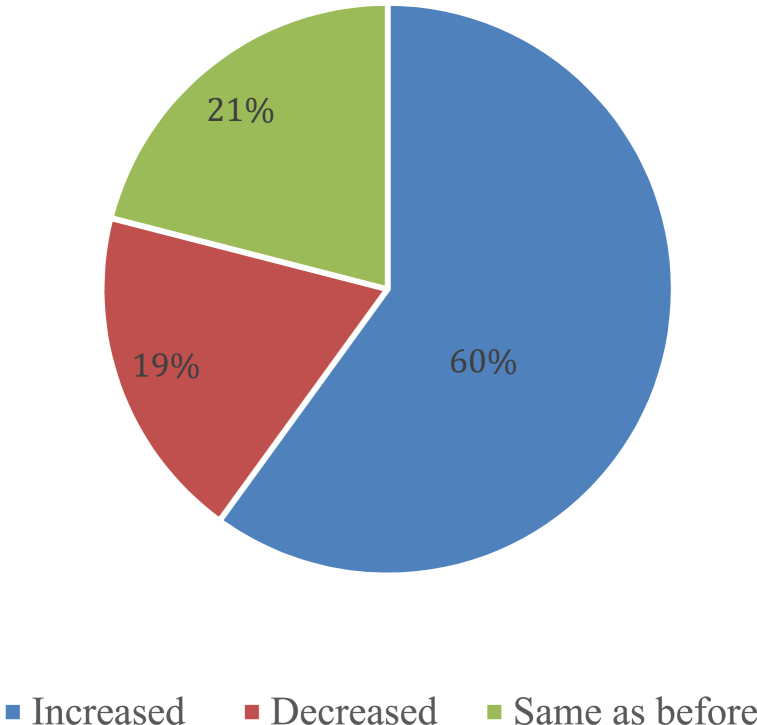
Changes in crop production due to Drought.

From Fig. 9, nearly 60 % of the farmers claim that the addition of deep ditches has improved the status of their crop yield. Once more, over 20 % of them are in the same situation as before, and nearly 19 % of them reported that deep irrigation has reduced agricultural productivity. The situation of crop production has also changed after the deep well irrigation system was implemented. However, there are a number of additional factors, such as fertilizers and medications, that contribute to the rising crop productivity. Crop intensification through crop diversity, cropping patterns, and etc. is another commonly employed adaptive approach in Bangladesh's Northwestern region, which has faced drought. Most farmers in this area have diversified their fields with crops like sugarcane, various types of pulse and oil crops, vegetables, and a variety of fruit crops including mango, jujube, and etc. to fend off drought. In contrast, Northwestern farmers frequently use the profitable combination of growing rice and mangoes as an adaptation approach [46].

**Fig. 10:**
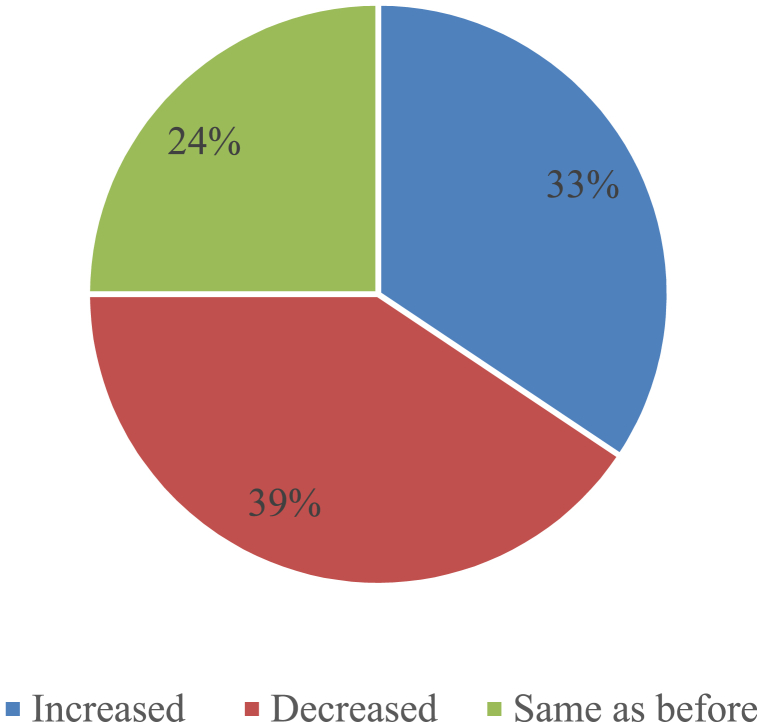
Cost status after switching crop production.

Fig. 10 depicts, the approximately 39 % of the participants reported a drop in production costs after transitioning from cultivating paddy or wheat to alternative crops such as pulses, nuts, or other water-efficient crops. Once again, about 36 % of respondents reported an increase in their costs, while approximately 25 % said that their cost status remained unchanged after switching crops. Various crop varieties exhibit varying water requirements and cultivation costs due to their distinct processes. Drought poses challenges for rural inhabitants and jeopardizes the agricultural, forestry, and livestock sectors. Most farmers in this area are cultivating a diverse range of crops to alleviate the impact of the drought. The crops cultivated in these fields encompass sugarcane, many types of legumes and oil crops, vegetable crops, as well as numerous types of fruit crops including mango and jujube. Notably, these fields practice the simultaneous cultivation of two different crops within the same area. Common adaption tactics employed in the Northwestern region of Bangladesh include the utilization of rice and mango. These measures not only mitigate the decrease in production caused by drought, but they also yield financial advantages for the farmers [46].

### 3.4 Analysis on effects on livelihood of people

**Fig. 11:**
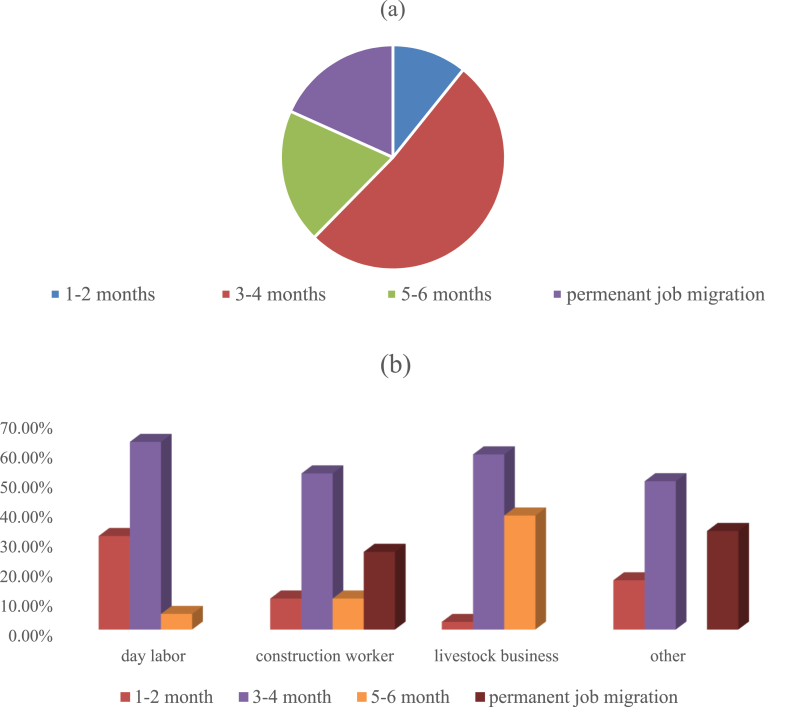
Migration of jobs in other sectors due to production loss (a) percentage of migration, (b) migration of jobs.

Fig. 11 illustrates that a majority of the farmers, including about half of the respondents, engage in seasonal migration for a duration of approximately 3–4 months. A mere 11 % of farmers engage in seasonal migration for a duration of 1–2 months in any given year. Approximately 20 % of farmers engage in seasonal migration for a duration of 5–6 months. Approximately 18.3 % of the farmers have permanently transitioned from agricultural work to other occupations. The remaining individuals engage in seasonal migration to sustain their regular revenue stream. These enduring alterations are a consequence of both the water issue and budgetary difficulties. Essentially, farmers engage in seasonal migration during periods of drought to adapt to the adverse climatic conditions. Out of these migratory individuals, around 59 % engage in livestock business. This movement is enabling farmers to financially maintain their livelihood during the drought season, when irrigation is more expensive and production is more challenging. Individuals relocate permanently due to the adverse impact of frequent and severe droughts on their financial resources and prospects in the surveyed region. Unemployment among agricultural laborers is caused by unproductive land. Currently, they engage in daily job. Even during the most challenging periods, farmers often transition to different professions and relocate to nearby towns or cities in order to secure employment and provide for their families. Seasonal migration occurs during adverse environmental conditions [46]. Local seasonal agricultural laborers and freshwater fishers sometimes encounter economic instability due to the nature of their profession. Seasonal migration is also caused by the decline of agriculture. Due to the crop's failure to yield the expected profits, agricultural investments incur financial losses due to both environmental and non-environmental factors. Most of the farmers, almost half of the respondents, engage in seasonal migration for almost for 3–4 month. Very few farmers, almost 11 %, engage in seasonal 22 migration for 1–2 months in any particular year. Approximately 20 % of the farmers engage in seasonal migration for 5–6 months. Almost 18.3 % of the farmers had done permanent job migration from agriculture. And rest of them are practicing seasonal migration for maintaining their normal income flow. These permanent changes are the result of water crisis and financial problems. Essentially, the farmers engage in seasonal migration in the drought period in order to cope with the unfavorable climatic condition. Of these seasonal migrants almost 59 % are migrated to livestock business. This migration is helping the farmers to provide monetary support to their livelihood in the drought season when irrigation is costlier and cultivation is harder. People move permanently because significant drought occurrences reduce or eliminate income and opportunity in the study area. Agricultural laborers are unemployed due to unproductive land. At this time, they work daily. Farmers shift careers and move to adjacent towns or cities to find work to support their family, even in the worst of times. Seasonal migration happens in harsh conditions Habiba et al. (2022). Seasonal agricultural laborers and freshwater fishers who work locally often face seasonal income uncertainty. Loss of agriculture also drives seasonal migration. Because the crop fails to provide predicted returns, agricultural investments lose money for environmental and non-environmental reasons. In their destinations, seasonal migrants learn supplementary occupations like rickshaw pulling, construction, and agriculture [47].

**Fig. 12:**
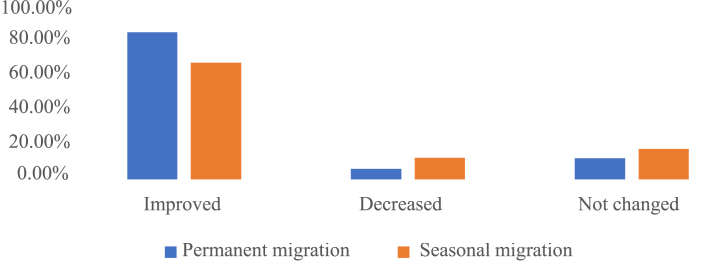
Change in living standard by job migration.

It is estimated that 80 percent of permanently relocated farmers have seen an improvement in their standard of life, while only roughly 65 percent of seasonally migrating farmers have seen such an improvement (Fig. 12). In times of severe drought, many farmers abandon their careers and move their families to a nearby city or hamlet in the hope of finding work that will allow them to provide for their families' most fundamental needs. In times of extreme hardship, people will migrate seasonally across this region [48]. Those who are most likely to be affected by unstable seasonal income are subsistence farmers, fishers, and agricultural laborers who must travel great distances from where they were born in order to make a living during times of seasonal instability.

## 4 Conclusion

Factors such as NDVI, NDWI, NDMI, Rainfall, VCI, TCI, and VHI were used to assess the Drought scenario in this analysis. By reducing the proportion of areas experiencing mild or no drought, the VCI, TCI, and VHI all signal a rise in areas experiencing severe or extreme drought. Extreme drought increased by 5 % (VCI), 11 % (TCI), and 7 % (VHI) by replacing no-drought areas in the relevant years. Vegetated land has also shrunk when compared to historic norms. The local climate is likewise warming gradually. There has been loss of agricultural output due to increased drought severity. 15 % of agricultural productivity has improved over the past. According to 39 % of respondents, moving to alternative crops reduced their production costs. The majority of farmers, about half of the respondents, migrate seasonally for three to four months. Very few farmers, approximately 11 %, engage in seasonal 22 migration for 1–2 months each year. Almost 18.3 % of farmers have made a permanent employment transition from agriculture whereas 80 % permanently relocated farmers reported an increase in their quality of life. Vegetated land has also shrunk when compared to historic norms. The local climate is likewise warming gradually. Irrigation and rain are the two most important factors in the region's agriculture. Loss of agricultural output due to irrigation water shortage. The majority of farmers have shifted to water-efficient crop varieties. For this reason, they relocate their workplace. During the severe dry season, most farmers switch to auto driving and labor. The majority of farmers in the study claimed that leaving their jobs and moving permanently to another occupation enhanced their standard of living. These extreme effects of drought have a wide range of consequences for the local population. Drought assessment and the changes in agricultural output and life standard that arise from it are key tools for coping with the devastating consequences of drought on agriculture and people's quality of life while also fostering sustainable development. These indices may be used to determine which areas are most at risk of experiencing drought, how much of an impact drought has on agricultural activity, and how successful mitigation strategies have been. Multiple research directions lie ahead for this area. Additional research directions may include the creation and refinement of these indices for use in drought vulnerability assessment. Improving the precision of existing indexes or creating whole new ones are both possibilities. Using these indices to create drought mitigation techniques that reduce agricultural output and livelihood losses is another possible direction for further study. This may involve optimizing a brand-new irrigation system, selecting drought-resistant plant varieties, collecting and storing rainfall for use in agriculture, and researching the most effective strategies for growing crops in dry environments.

## CRediT authorship contribution statement

**Anika Tahasin:** Writing – original draft, Visualization, Methodology, Investigation, Formal analysis, Conceptualization. **Mafrid Haydar:** Writing – review & editing, Supervision, Methodology, Formal analysis, Data curation, Conceptualization. **Md. Sabbir Hossen:** Writing – review & editing, Visualization, Validation, Data curation. **Halima Sadia:** Writing – review & editing, Visualization, Methodology, Formal analysis.

## Ethical approval

The authors declare No ethical approval required. Ethical approval for this type of study is not required by our institute.

## Data and code availability

Data will be made available on request.

## Funding

None.

## Declaration of competing interest

The authors declare that they have no known competing financial interests or personal relationships that could have appeared to influence the work reported in this paper.

## References

[1] Islam A.R.T., Shen S., Hu Z., Rahman M.A. (2017). Drought hazard evaluation in boro paddy cultivated areas of western Bangladesh at current and future climate change conditions. Adv. Meteorol..

[2] Shahid S., Behrawan H. (2008). Drought risk assessment in the western part of Bangladesh. Nat. Hazards.

[3] Shahid S. (2010). Recent trends in the climate of Bangladesh. Clim. Res..

[4] Zhou K., Li J., Zhang T., Kang A. (2021). The use of combined soil moisture data to characterize agricultural drought conditions and the relationship among different drought types in China. Agric. Water Manag..

[5] Hanadé Houmma I., El Mansouri L., Gadal S., Garba M., Hadria R. (2022). Modelling agricultural drought: a review of latest advances in big data technologies. Geomatics, Nat. Hazards Risk.

[6] Das A.C., Shahriar S.A., Chowdhury M.A., Hossain M.L., Mahmud S., Tusar M.K., Ahmed R., Salam M.A. (2023). Assessment of remote sensing-based indices for drought monitoring in the north-western region of Bangladesh. Heliyon.

[7] World Meteorological Organization (2021). State of the global climate 2020. https://library.wmo.int/index.php?lvl=notice_display&id=21880#.YHg0ABMzZR0.

[8] Adler R.W. (2010). Drought, sustainability, and the law. SSRN Electron. J..

[9] Carrão H., Naumann G., Barbosa P. (2016). Mapping global patterns of drought risk: an empirical framework based on sub-national estimates of hazard, exposure and vulnerability. Global Environmental Change-Human and Policy Dimensions.

[10] Dai A. (2012). Increasing drought under global warming in observations and models. Nat. Clim. Change.

[11] Allen C.D., Macalady A.K., Chenchouni H., Bachelet D., McDowell N., Vennetier M., Kitzberger T., Rigling A., Breshears D.D., Ted Hogg E.H., Gonzalez P., Fensham R., Zhang Z., Castro J., Demidova N., Lim J.H., Allard G., Running S.W., Semerci A., Cobb N. (2010). A global overview of drought and heat-induced tree mortality reveals emerging climate change risks for forests. For Ecol Manage.

[12] Lewis S.L., Brando P.M., Phillips O.L., Van Der Heijden G.M.F., Nepstad D. (2011). The 2010 Amazon drought. Science.

[13] Bhardwaj K., Shah D., Aadhar S., Mishra V. (2020). Propagation of meteorological to hydrological droughts in India. J. Geophys. Res. Atmos..

[14] Rahman M.M., Rahaman M.M. (2018). Impacts of Farakka barrage on hydrological flow of Ganges river and environment in Bangladesh. Sustainable Water Resources Management.

[15] Mondol M.A.H., Zhu X., Dunkerley D., Henley B.J. (2021). Observed meteorological drought trends in Bangladesh identified with the Effective Drought Index (EDI). Agric. Water Manag..

[16] Al Kafy A., Bakshi A., Saha M., Al Faisal A., Almulhim A.I., Rahaman Z.A., Mohammad P. (2023). Assessment and prediction of index based agricultural drought vulnerability using machine learning algorithms. Sci. Total Environ..

[17] Zakaria M., Abdullah Aziz M., Hossain M.I., Ismail Hossain M., Md Farhat Rahman N. (2014). Effects of rainfall and maximum temperature on aman rice production of Bangladesh: a case study for last decade remote sensing and GIS in agriculture view project I'm working in JIRCAS project view project effects of rainfall and maximum temperature on Am. International Journal of Scientific & Technology Research.

[18] Ashik Ali Murad M., Hossain Mishu M., Tasnim J., Zubayer S., Murad M., Hossain M., Jahan T., Tahasin A. (2019). Irrigation water crisis and changes in agricultural practice in barind area A study in Godagari upazila 4 publications 2 citations see profile irrigation water crisis and changes in agricultural practice in barind area A study in Godagari upazila. https://www.researchgate.net/publication/358046075.

[19] Haydar M., Hossain Rafi A., Sadia H., Tanvir Hossain M. (2024). Data driven forest fire susceptibility mapping in Bangladesh. Ecol Indic.

[20] Sadia H., Sarkar S.K., Haydar M. (2023). Soil erosion susceptibility mapping in Bangladesh. Ecol Indic.

[21] Maes W.H., Steppe K. (2012). Estimating evapotranspiration and drought stress methylation and chromatin patterning with ground-based thermal remote sensing in agriculture: a review. J. Exp. Bot..

[22] West H., Quinn N., Horswell M. (2019). Remote sensing for drought monitoring & impact assessment: progress, past challenges and future opportunities. Remote Sens. Environ..

[23] Haile G.G., Tang Q., Hosseini-Moghari S.M., Liu X., Gebremicael T.G., Leng G., Kebede A., Xu X., Yun X. (2020). Projected impacts of climate change on drought patterns over east Africa. Earth's Future.

[24] Bento V.A., Gouveia C.M., DaCamara C.C., Trigo I.F. (2018). A climatological assessment of drought impact on vegetation health index. Agric. For. Meteorol..

[25] Adhikary S.K., Das S.K., Saha G.C., Chaki T. (2013). Proceedings - 20th International Congress on Modelling and Simulation.

[26] Kogan F.N. (1995). Application of vegetation index and brightness temperature for drought detection. Adv. Space Res..

[27] Kogan F.N. (2001). Operational space technology for global vegetation assessment. Bull. Am. Meteorol. Soc..

[28] Kogan F.N. (1990). Remote sensing of weather impacts on vegetation in non-homogeneous areas. Int. J. Rem. Sens..

[29] Guha S., Govil H., Diwan P. (2020). Monitoring LST-NDVI relationship using premonsoon landsat datasets. Adv. Meteorol..

[30] Kumar A., Bhattacharya T., Shaikh W.A., Roy A. (2024). Sustainable soil management under drought stress through biochar application: immobilizing arsenic, ameliorating soil quality, and augmenting plant growth. Environ. Res..

[31] Piao S., Ciais P., Huang Y., Shen Z., Peng S., Li J., Zhou L., Liu H., Ma Y., Ding Y., Friedlingstein P., Liu C., Tan K., Yu Y., Zhang T., Fang J. (2010). The impacts of climate change on water resources and agriculture in China. Nature.

[32] Vicente-Serrano S.M., Quiring S.M., Peña-Gallardo M., Yuan S., Domínguez-Castro F. (2020). A review of environmental droughts: increased risk under global warming?. Earth Sci. Rev..

[33] Al Faisal A., Kafy A.A., Al Rakib A., Akter K.S., Jahir D.M.A., Sikdar M.S., Ashrafi T.J., Mallik S., Rahman M.M. (2021). Assessing and predicting land use/land cover, land surface temperature and urban thermal field variance index using Landsat imagery for Dhaka Metropolitan area. Environmental Challenges.

[34] Bindajam A.A., Mallick J., Talukdar S., Shahfahad, Shohan A.A.A., Rahman A. (2022). Modeling the spatiotemporal heterogeneity of land surface temperature and its relationship with land use land cover using geo-statistical techniques and machine learning algorithms. Environ. Sci. Pollut. Control Ser..

[35] Xulu S., Peerbhay K., Gebreslasie M., Ismail R. (2018). Drought influence on forest plantations in Zululand, South Africa, using MODIS time series and climate data. Forests.

[36] Kefalas G., Lattas P., Xofis P., Lorilla R.S., Martinis A., Poirazidis K. (2018). The use of vegetation indices and change detection techniques as a tool for monitoring ecosystem and biodiversity integrity. International Journal of Sustainable Agricultural Management and Informatics.

[37] Alharbi R.S., Nath S., Faizan O.M., Hasan M.S.U., Alam S., Khan M.A., Bakshi S., Sahana M., Saif M.M. (2022). Assessment of drought vulnerability through an integrated approach using AHP and geoinformatics in the kangsabati river basin. J. King Saud Univ. Sci..

[38] Ermida S.L., Soares P., Mantas V., Göttsche F.M., Trigo I.F. (2020). Google earth engine open-source code for land surface temperature estimation from the landsat series. Rem. Sens..

[39] Fleming-Muñoz D.A., Whitten S., Bonnett G.D. (2023). The economics of drought: a review of impacts and costs. Aust. J. Agric. Resour. Econ..

[40] Tian M., Wang P., Khan J. (2016). Drought forecasting with vegetation temperature condition index using arima models in the guanzhong plain. Rem. Sens..

[41] Kumar S., Tripathi S., Singh S.P., Prasad A., Akter F., Syed M.A., Badri J., Das S.P., Bhattarai R., Natividad M.A., Quintana M., Venkateshwarlu C., Raman A., Yadav S., Singh S.K., Swain P., Anandan A., Yadaw R.B., Mandal N.P., Verulkar S.B., Kumar A., Henry A. (2021). Rice breeding for yield under drought has selected for longer flag leaves and lower stomatal density. J. Exp. Bot..

[42] Gidey E., Dikinya O., Sebego R., Segosebe E., Zenebe A. (2018). Analysis of the long-term agricultural drought onset, cessation, duration, frequency, severity and spatial extent using Vegetation Health Index (VHI) in Raya and its environs, Northern Ethiopia. Environmental Systems Research.

[43] Dutta D., Kundu A., Patel N.R., Saha S.K., Siddiqui A.R. (2015). Assessment of agricultural drought in Rajasthan (India) using remote sensing derived vegetation condition index (VCI) and standardized precipitation index (SPI). Egyptian Journal of Remote Sensing and Space Science.

[44] Zhao X., Xia H., Liu B., Jiao W. (2022). Spatiotemporal comparison of drought in shaanxi–gansu–ningxia from 2003 to 2020 using various drought indices in google earth engine. Rem. Sens..

[45] Orhan O., Ekercin S., Dadaser-Celik F. (2014). Use of Landsat land surface temperature and vegetation indices for monitoring drought in the Salt Lake Basin Area, Turkey. Sci. World J..

[46] Habiba U., Shaw R., Takeuchi Y. (2012). Farmer's perception and adaptation practices to cope with drought: perspectives from Northwestern Bangladesh. Int. J. Disaster Risk Reduc..

[47] Huq F.F. (2020). Impact of groundwater drought on domestic water use in barind tract, Bangladesh, J. Wat. Env. Sci.

[48] Habiba U., Shaw R., Takeuchi Y. (2012). Farmer's perception and adaptation practices to cope with drought: Perspectives from Northwestern Bangladesh. Int. J. Disaster Risk Reduc..

